# New and Emerging Therapeutic Drugs for the Treatment of Pulmonary Arterial Hypertension: A Systematic Review

**DOI:** 10.7759/cureus.68117

**Published:** 2024-08-29

**Authors:** Amir Rasheed, Shadab Aslam, Hafiz Zeeshan Sadiq, Salamat Ali, Rizwana Syed, Binay K Panjiyar

**Affiliations:** 1 Internal Medicine, Aziz Bhatti Shaheed Teaching Hospital, Gujrat, PAK; 2 Internal Medicine, Jinnah Hospital, Lahore, PAK; 3 Medicine, Aziz Bhatti Shaheed Teaching Hospital, Gujrat, PAK; 4 General Surgery, Aziz Bhatti Shaheed Teaching Hospital, Gujrat, PAK; 5 Internal Medicine, Apollo Institute of Medical Sciences and Research, Chittoor, Chittoor, IND; 6 Research, Ventolini's Lab, Texas Tech University Health Sciences Center, Odessa, USA; 7 Global Clinical Scholars Research Training, Harvard Medical School, Boston, USA; 8 Internal Medicine, California Institute of Behavioral Neurosciences & Psychology, Fairfield, USA

**Keywords:** primary pulmonary hypertension, pulmonary hypertension, pulmonary hypertension class 1, treatment of pulmonary arterial hypertension, pulmonary arterial hypertension

## Abstract

Pulmonary arterial hypertension (PAH) is a serious, progressive, and potentially fatal lung disease characterized by a gradual increase in mean pulmonary arterial pressure to over 20 mmHg at rest. The pathogenesis of PAH is multifactorial. It involves dynamic obstruction of the pulmonary vasculature through vasoconstriction, structural obstruction due to adverse vascular remodeling, and pathological obstruction caused by vascular fibrosis and stiffening, which reduces compliance. PAH often presents with vague initial symptoms and is frequently diagnosed at an advanced stage. The increased pulmonary arterial pressure leads to vascular remodeling, eventually resulting in right ventricular hypertrophy and failure. PAH is a rare condition with a median life expectancy of three years, underscoring the need for effective treatment alternatives. Several FDA-approved therapeutic options are available, including prostacyclin analogs (epoprostenol, iloprost, and treprostinil), the non-prostanoid IP receptor agonist selexipag, selective endothelin receptor antagonists (ERA) (ambrisentan, bosentan, and macitentan), phosphodiesterase 5 inhibitors (sildenafil and tadalafil), and the soluble guanylate cyclase (sGC) stimulator riociguat. Despite these advancements, current medications do not provide a permanent cure. This study presents an overview of current and emerging PAH therapies through a systematic literature review. It involved an analysis of nine studies and a review of 800 papers from reputable journals published between 2013 and June 2023. The research focused on drug effects on the six-minute walk distance (6-MWD) and associated side effects in randomized controlled trials. The review found that while udenafil, imatinib, racecadotril, sotatercept, anastrozole, riociguat, tacrolimus, and ralinepag were evaluated, imatinib was notably associated with adverse side effects. Conversely, udenafil, racecadotril, sotatercept, anastrozole, riociguat, tacrolimus, and ralinepag were found to be safe, well-tolerated, and effective in improving hemodynamic measures and 6-MWDs. This study aims to summarize the developing treatment options currently under clinical trials, highlighting the need for further trials before their application in clinical practice.

## Introduction and background

In the vast realm of pulmonary disorders, pulmonary arterial hypertension (PAH) has emerged as a particularly daunting adversary for clinicians. Defined by its insidious augmentation of pulmonary vascular resistance (PVR), PAH inexorably advances, often culminating in debilitating right-sided heart failure [1]. Such a progression invariably diminishes both the quality and span of an affected individual’s life [2]. Notwithstanding the commendable advances made by current FDA-approved therapeutic interventions, which have decidedly improved hemodynamic profiles and elevated patients’ daily life experiences, the global prognosis of PAH remains poor [3,4]. This manuscript delves into the intricacies of the treatment of PAH, seeking to summarize new and emergent therapeutic avenues, aiming to reshape the trajectory of this formidable condition. Notably, the median survival period persists under three years post-diagnosis, and none of the existing medications offer tangible survival benefits.

The diagnostic hallmarks of PAH, such as mean pulmonary arterial pressure exceeding 20 mmHg, which is above the 97.5th percentile, and PVR beyond 3 WU [5]. The WHO’s taxonomy further refines our understanding by stratifying pulmonary hypertension into five distinct groups. Group I (PAH) encompasses diverse etiologies, ranging from idiopathic origins and genetic mutations such as bone morphogenetic protein receptor 2 (BMPR2; the most common), AKL1, Endoglein, CVA1, SMAD9, and KCNK3 to drug-induced variants (including amphetamines, methamphetamines, cocaine, and fenfluramine-phentermine) and those interlinked with systemic conditions (such as connective tissue disorders, HIV infection, and congenital heart diseases) and PAH with features of venous/capillary involvement [6].

In the cellular and molecular strata, PAH is restricted by intricate pathophysiological transformations. Central to these is vascular remodeling, a cascade of changes that includes constriction and eventual obliteration of minuscule arterioles, thereby exacerbating PVR [2,7]. Such shifts are further compounded by dysfunctional endothelial and smooth muscle cells, leading to emblematic plexiform lesions in PAH. Furthermore, the hyperproliferation and misplaced migration of smooth muscle cells constrict luminal pathways, engendering heightened vascular pressure [8].

Clinically, the manifestations of PAH, albeit nonspecific, gravely impact the lives of patients. These symptoms, from exertional fatigue and dyspnea to syncope and persistent cough, underscore the pressing need for advanced diagnostic modalities, encompassing ECGs, arterial blood gas analyses, and, notably, right heart catheterization [9]. Amid the currently available treatments, agents, including prostacyclin analogs, non-prostanoid IP receptor agonists, and phosphodiesterase-5 inhibitors, have offered therapeutic reprieves [10]. However, the persistent quest for innovative therapies underscores the existing treatment gaps. This manuscript, through a systematic review, endeavors to highlight both newly introduced therapeutics and those currently undergoing rigorous clinical evaluation.

## Review

Methodology

**Figure 1:**
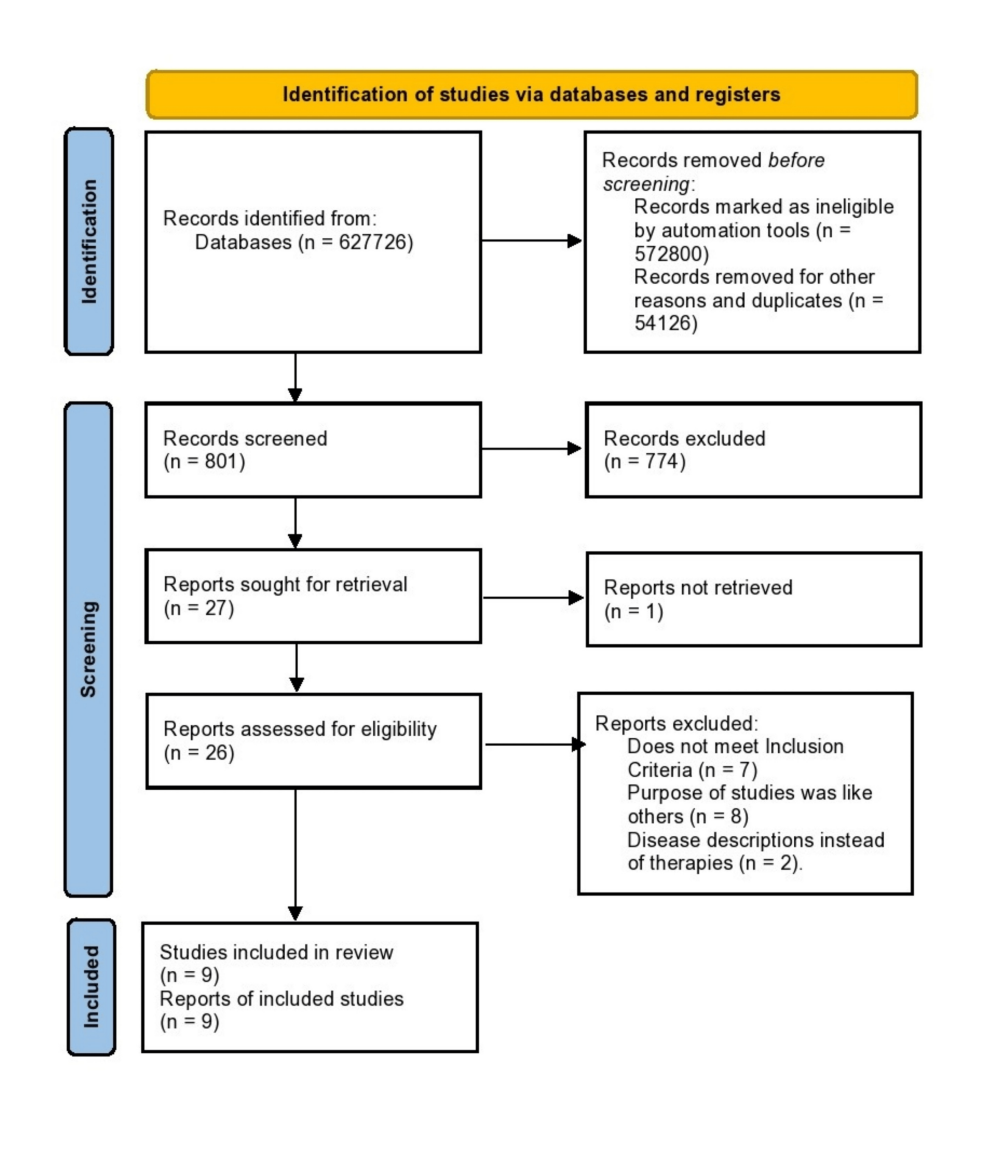
PRISMA flow diagram illustrating the search strategy and study selection process for the systematic review PRISMA, Preferred Reporting Items for Systematic reviews and Meta-Analyses

This review focuses on clinical investigations related to therapies for PAH, excluding research involving animal subjects. Adhering to the Preferred Reporting Items for Systematic reviews and Meta-Analyses (PRISMA) guidelines, this review did not require ethical approval as the data were sourced exclusively from published scientific papers. A comprehensive search strategy was employed, yielding 54,756 results from PubMed (including Medline) and 573,000 results from Google Scholar. Automation tools were used to narrow the search, leading to the exclusion of 54,126 PubMed records and 572,800 Google Scholar results from the past decade. After initial screening, 801 records were reviewed, resulting in the exclusion of 774 ineligible entries. Of the 27 reports identified, 26 were retrieved and assessed for eligibility, with 17 records excluded after thorough scrutiny. Ultimately, nine records were included in the systematic review (Figure 1).

Systematic Literature and Study Selection

A comprehensive search for pertinent publications was performed using PubMed, encompassing Medline and Google Scholar. In PubMed, we also scrutinized studies cited in editorials and reviews. We compiled a list of abstracts independently evaluated for inclusion based on predetermined criteria. These criteria encompassed topics related to pulmonary hypertension or PAH, drugs, treatments, and therapies. The exclusion criteria were animal studies and research involving pediatric populations. Four independent reviewers conducted a dual review to ensure accuracy and consistency, and any discrepancies were resolved through discussion.

Inclusion and Exclusion Criteria

We developed inclusion and exclusion criteria, which are detailed in Table 1.

**Table 1:** Inclusion and exclusion criteria

S. no.	Inclusion criteria	Exclusion criteria
1	Human studies	Animal studies
2	Studies from 2013 to 2023	Studies before 2013
3	English text	Non-English text
4	All genders	
5	Adults age 18 or higher	Ages below 18
6	Free papers	Paid papers

Search Strategy

We utilized the Population, Intervention/Condition, Comparison/Control, and Outcome (PICO) criteria and conducted a thorough literature review using databases such as Google Scholar and PubMed (including Medline). MeSH terms were employed in PubMed and Google Scholar to develop a comprehensive search strategy, as detailed in Table 2.

**Table 2:** Search strategy employed in the study

Database	Search strategy	Search results
PubMed (including Medline)	Treatment or management or therapy and pulmonary hypertension or pulmonary arterial hypertension	54,756
Google Scholar	Pulmonary arterial hypertension AND treatment	573,000

Quality Appraisal

To ensure the credibility of the selected articles, we employed various quality assessment methods. For systematic reviews and meta-analyses, we followed the PRISMA checklist. Randomized clinical trials were evaluated using the Cochrane Risk of Bias Tool. Non-randomized clinical studies were assessed with the Newcastle-Ottawa Scale, and qualitative investigations were reviewed using the Critical Appraisal Skills Programme (CASP) checklist, as detailed in Table 3. To maintain classification clarity and avoid ambiguity, we utilized the Scale for the Assessment of Narrative Review Articles (SANRA) for evaluating narrative review articles.

**Table 3:** Quality appraisal tools used in the study PRISMA, Preferred Reporting Items for Systematic reviews and Meta-Analyses; SANRA: Scale for the Assessment of Narrative Review Articles

Quality appraisal tool	Type of studies
Cochrane Risk of Bias Tool	Randomized control trials
New Castle-Ottawa Scale	Non-randomized control trials and observational studies
PRISMA Checklist	Systematic reviews
SANRA Checklist	Other studies without a clear methodological selection

Results

The summary of the results from the selected papers is presented in Table 4.

**Table 4:** Summary of results from selected papers BMPR2, bone morphogenetic protein receptor 2; ERA: endothelial receptor antagonist; IMPRES: Imatinib in Pulmonary Arterial Hypertension, a Randomized Efficacy Study; MWD, minute walking distance; PAH, pulmonary arterial hypertension; PATENT, Pulmonary Arterial Hypertension Soluble Guanylate Cyclase–Stimulator Trial; PDE-5i: phosphodiesterase 5 inhibitor, RCT, randomized controlled trial

Author and year	Country	Study design	Database used	Conclusion
Dhoble et al. (2022) [10]	Netherlands	Review article	-	PAH is an incurable disease; further exploration of therapeutic options is needed.
Chang et al. (2019) [11]	USA	RCT	A total of 63 patients with PAH were randomized into either the placebo group or the udenafil group.	Udenafil was found to improve exercise capacity in patients with PAH, using 6-MWD.
Frost et al. (2015) [12]	USA	RCT	This extension study of the IMPRES trial included 202 patients. Of these, 78 were randomized into the placebo group, and 66 were randomized into the imatinib group.	Imatinib improved exercise tolerance but had severe adverse effects; the risks outweighed the benefits.
Hobbs et al. (2019) [13]	UK	RCT	Twenty-one PAH patients stable on PDE-5i were randomized in a 2:1 ratio to receive either racecadotril or placebo. Outcomes were assessed both acutely and after 14 days.	Racecadotril may have therapeutic utility, but large-scale prospective trials are necessary.
Humbert et al. (2021) [14]	UK	RCT	A total of 106 adults receiving background therapy for PAH were randomized into either the treatment or placebo groups.	Sotatercept was associated with improvements in PVR in patients receiving background therapy.
Kawut et al. (2017) [15]	USA	RCT	A total of 18 patients receiving background therapy for PAH were randomized into either the treatment or placebo groups.	Anastrazole had no effect on biomarkers or quality of life but improved the six-minute walking distance.
Rubin et al. (2015) [16]	Europe	RCT	In the PATENT-2 study, 396 patients who had completed the PATENT-1 study were eligible and were divided into three groups: 231 patients received a maximum of 2.5 mg riociguat, 56 patients received a maximum of 1.5 mg riociguat, and 109 patients were assigned to the placebo group.	Riociguat was safe and effective for long-term use, either alone or with ERA and prostanoids.
Spiekerkoetter et al. (2017) [17]	Europe	RCT	In a 16-week placebo-controlled phase 2a trial, 23 patients with PAH were randomized to receive either a placebo or the treatment.	Low-level tacrolimus is safe, well-tolerated, and associated with increased BMPR2 levels and improved six-minute walk distance.
Torres et al. (2019) [18]	Europe	RCT	Sixty-one PAH patients receiving standard background therapy were randomized in a 2:1 ratio to either ralinepag or placebo groups.	Ralinepag has a tolerable safety profile and is associated with reduced PVR.

Discussion

Various FDA-approved drugs are available for treating PAH, categorized by their mechanisms of action into prostacyclin analogs, receptor agonists, PDE inhibitors, and endothelial receptor antagonists. Within the prostacyclin analog and receptor agonist class, FDA-approved drugs include epoprostenol, treprostinil, and iloprost [10,19]. For PDE inhibitors, FDA-approved options are sildenafil, tadalafil, and vardenafil [10,20]. In the endothelin receptor antagonist (ERA) class, approved drugs include bosentan, ambrisentan, and macitentan [10,21]. The findings of this systematic review are summarized below.

Udenafil

Udenafil is a novel phosphodiesterase-5 (PDE-5) inhibitor, notable for its efficacy and safety profile comparable to other PDE-5 inhibitors and a longer half-life than sildenafil, allowing for sustained vasodilation up to 12 hours post-administration [22,23]. In a double-blinded, placebo-controlled phase 2a trial, a 50 mg dose of udenafil was observed to produce a greater reduction in PVR compared to a 100 mg dose at baseline, although the cardiac index remained stable at most time points. This finding suggested that the 50 mg dose should be used in the phase 2b trial [24].

In the subsequent 16-week phase 2b clinical trial, 63 PAH patients were randomly assigned to receive either a placebo or 50 mg of udenafil. The results indicated a mean placebo-corrected improvement of 25 meters in the six-minute walk distance (6-MWD) (p = 0.0873). Among patients with prior ERA therapy, the treatment effect was more pronounced at 34 meters (p = 0.0460). However, no significant changes were noted in the Borg dyspnea score [11]. The study concluded that udenafil was well-tolerated and had a positive impact on 6-MWD, which might enhance patients’ quality of life. Further extended studies were recommended to confirm these findings [11].

Imatinib

Imatinib is an oral inhibitor of protein kinases, which has been considered to be a potential factor in the development of pulmonary hypertension [25]. In a prior 24-week randomized controlled trial known as the Imatinib in Pulmonary Arterial Hypertension, a Randomized Efficacy Study (IMPRES), patients diagnosed with PAH who did not respond to at least two PAH-specific therapies exhibited improvements in hemodynamics, exercise capacity, and PVR [26].

To assess the long-term safety and efficacy of imatinib, an open-label extension study of the IMPRES trial was conducted, spanning up to 204 weeks and concluding on April 16, 2014. Eligible participants for this extension were those who had completed the original IMPRES trial [12]. Of the 202 patients initially enrolled in IMPRES, 66 received imatinib, and 78 received a placebo in the extension study. Ultimately, 93.8% (135 out of 144) of the participants withdrew from the extension trial, with the primary reasons being administrative issues (e.g., sponsor termination; 32.6%) and adverse events (31.3%) [12]. Only nine individuals completed the extension study. Unexpected and severe adverse events were frequent, including 17 fatalities either during the extension study or within 30 days after its termination, as well as six cases of subdural hematomas [12]. While those patients who tolerated imatinib and participated in the extension for an extended period experienced improvements in functional class and walking distance, most participants discontinued both the medication and the trial. The occurrence of severe adverse events, substantial side effects, and a high dropout rate constrain imatinib’s potential effectiveness in treating PAH. These risks outweigh any potential benefits in terms of hemodynamics and walking distance observed in individuals who can continue taking the medication. Consequently, the off-label use of this substance for PAH treatment is not recommended [12].

Sotarercept

Sotatercept is an innovative fusion protein designed to bind activating and growth-differentiating factors to restore a balance between growth-promoting and inhibitory signaling pathways [27]. Previous evaluations of sotatercept encompassed studies involving healthy volunteers, as well as patients with conditions such as chemotherapy-induced anemia, myelodysplastic syndromes, end-stage renal disease, multiple myeloma, β-thalassemia, and myelodysplastic syndromes [28-33].

In the context of a 24-week randomized multicenter placebo-controlled trial known as the PULSAR trial, 106 patients already receiving background therapy for PAH were randomly assigned to one of three groups. These groups received either 0.3 mg/kg of sotatercept every three weeks, 0.7 mg/kg of sotatercept every three weeks, or a placebo. The primary endpoint of this trial was the change in PVR from baseline to week 24 [14]. The three groups shared comparable baseline characteristics.

Regarding the change in PVR from baseline to week 24, the sotatercept 0.3 mg/kg group demonstrated a least-squares mean difference of 145.8 dyn·s·cm⁻⁵ compared to the placebo group (95% CI, 241.0 to 50.6; p = 0.003). Similarly, the sotatercept 0.7 mg/kg group exhibited a least-squares mean difference of 239.5 dyn·s·cm⁻⁵ compared to the placebo group (95% CI, 329.3 to 149.7; p < 0.001). Additionally, the change in the 6-MWD from baseline to week 24 showed a least-squares mean difference of 29.4 meters for the sotatercept 0.3 mg/kg group compared to the placebo group (95% CI, 3.8 to 55.0). The sotatercept 0.7 mg/kg group had a difference in least-squares mean of 21.4 meters compared to the placebo group (95% CI, 2.8 to 45.7) [14]. Sotatercept was also associated with a decrease in N-terminal pro-B-type natriuretic peptide levels [14].

The use of sotatercept in conjunction with background therapy demonstrated improvements in PVR. However, the need for larger-scale trials to further evaluate its efficacy was emphasized [14].

Anastrazole

Anastrazole is classified as an aromatase inhibitor that impedes the conversion of androgen into estrogen. It demonstrates the capacity to lower pulmonary arterial pressure, indices associated with right ventricular hypertrophy, and alterations in pulmonary vasculature [34,35]. Anastrozole was the subject of investigation in a randomized, double-blind, placebo-controlled study conducted in two distinct locations, involving patients with PAH who were concurrently receiving background medication. In this study, a total of 18 individuals diagnosed with PAH were randomly assigned in a 2:1 ratio to receive either anastrozole 1 mg or a placebo that matched the active medication. The study’s co-primary endpoints were the percentage change in 17b-estradiol levels (E2) from baseline and the tricuspid annular plane systolic excursion (TAPSE) after a three-month period. While no significant difference was observed in TAPSE measurements, anastrozole exhibited a profound reduction in E2 levels compared to the placebo group (percent change: 240%; IQR, 261 to 226% vs. 24%; IQR, 214 to 14%; p = 0.003). Additionally, anastrozole led to a substantial improvement in the 6-MWD (median change = 126 meters) (anastrozole group, 8%; IQR, 2 to 17% vs. placebo, 22%; IQR, 27 to 11%; p = 0.042) in comparison to the placebo [15].

Notably, anastrozole had no discernible impact on the functional class, health-related quality of life, or circulating biomarkers. However, it effectively reduced E2 levels, although TAPSE remained unchanged and the 6-MWD was enhanced. The treatment was well-tolerated and safe, although further phase 2 trials are warranted for additional evaluation [15].

Ralinepag

Ralinepag is an orally administered selective non-prostanoid prostacyclin receptor agonist characterized by a 24-hour half-life [36]. To evaluate its safety, tolerability, and efficacy, a randomized placebo-controlled trial was conducted in patients with PAH who were either on mono or dual background therapy [18]. In this trial, 61 PAH patients receiving conventional treatment, including mono or dual PAH-targeted background medications, were randomly assigned in a 2:1 ratio to receive ralinepag (n = 40) or placebo (n = 21). The initial dose of ralinepag was set at 10 g twice daily, and over the subsequent nine-week dose-titration phase, the dosage was increased as tolerated, up to a maximum total daily dose of 600 g (300 g twice daily). The primary efficacy endpoint was the absolute difference between baseline and week 22 in PVR. Secondary endpoints encompassed other hemodynamic measures, the 6-MWD, safety and tolerability, and the percentage change in PVR from baseline.

Results indicated a substantial reduction in PVR by 163.9 dyn·s·cm⁻⁵ with ralinepag, in contrast to a negligible increase of 0.7 dyn·s·cm⁻⁵ observed in the placebo group (p = 0.02). Furthermore, the least-squares mean improvement in PVR from baseline with ralinepag was 29.8% (p = 0.03). In terms of the 6-MWD, ralinepag led to an increase of 36.2 meters from baseline, while the placebo group demonstrated a 29.4-meter increase (p = 0.90). Safety-wise, ralinepag patients experienced serious adverse events at a rate of 10% as opposed to 29% in the placebo group. Additionally, 13% of ralinepag patients were part of the study dropouts, compared to 10% in the placebo group [18]. It is worth noting that further clinical and long-term studies are necessary to validate these findings and assess the broader clinical implications of ralinepag in PAH treatment.

Tacrolimus

The activation of the BMPR2 signaling pathway through tacrolimus (FK506) has been found to reverse occlusive vasculopathy in rodent models of PAH [37]. Previously, the use of low-dose tacrolimus (FK506) over a period of 12 months in three patients with end-stage PAH demonstrated improvements in heart failure symptoms, the 6-MWD, N-terminal pro-brain natriuretic peptide (NT-proBNP) levels, and increased BMPR2 expression in peripheral blood mononuclear cells (PBMCs) [38]. To assess the safety and tolerability of low-dose FK506 therapy in stable PAH patients with New York Heart Association functional class II/III symptoms, a randomized, double-blind, placebo-controlled, 16-week, single-center phase IIa trial was conducted. This trial involved three FK506 target levels (<2, 2-3, and 3-5 ng·mL−1) [17]. Out of the 23 patients randomly assigned to the trial, 20 successfully completed it. FK506 therapy was well tolerated, with nausea and diarrhea being the most commonly reported side effects. It is worth noting that BMPR2 expression in PBMCs was notably lower in PAH patients compared to healthy controls (n = 13; p = 0.005), but it increased following FK506 therapy [17].

Although certain patients exhibited improvements in the 6-MWD, serological markers related to heart failure, and echocardiographic measurements, these changes did not reach statistical significance [17]. Nevertheless, in specific subgroups of PAH patients, low-dose FK506 was well tolerated, leading to an elevation in BMPR2 expression. These findings support the initiation of a phase IIb efficacy trial to investigate the possibility of FK506 in PAH treatment additionally [17].

Riociguat

Riociguat represents a stimulant for soluble guanylate cyclase (sGC). Its mechanism of action involves sensitizing sGC to endogenous nitric oxide (NO), stabilizing the binding between NO and sGC, and directly activating sGC through a distinct binding site, independent of NO. Consequently, this leads to an elevation in cyclic GMP (cGMP) levels [39]. It has gained approval for its application in the treatment of PAH [3].

In the PATENT-1 study, conducted over a 12-week period (Pulmonary Arterial Hypertension Soluble Guanylate Cyclase-Stimulator Trial 1), riociguat demonstrated a significant enhancement in the 6-MWD for PAH patients when compared to a placebo group, with a least-squares mean difference of 36 meters (95% CI: 20-52 meters, p < 0.0001) [4]. Additionally, riociguat exhibited improvements in PVR (p < 0.0001), WHO functional class (p < 0.0033), time to clinical deterioration (p = 0.0046), and Borg dyspnea score (p = 0.002). Furthermore, it was generally well tolerated [4].

The PATENT-2 study involved 396 participants and aimed to assess the safety and tolerability of riociguat over an extended duration. Of the 405 patients who completed the PATENT-1 trial, 396 (98%) were enrolled in the PATENT-2 study, and these two groups exhibited comparable baseline characteristics. Within this study, 231 individuals were placed in the riociguat 2.5 mg maximum dose group, 56 in the riociguat 1.5 mg maximum dose group, and 109 in the placebo group. The findings of the PATENT-2 study indicated that the improvements observed in the WHO functional class and 6-MWD during the 12-week PATENT-1 trial were sustained for a period of one year. At the 12-week mark of the PATENT-2 study, the group receiving the maximum dose of riociguat (2.5 mg) experienced a 52-meter increase in the 6-MWD (after six months of active treatment). After one year of therapy, patients achieved a 51-meter improvement, and a substantial proportion, 61% and 33%, respectively, displayed stabilization and enhancement in the WHO functional class. These findings affirm that riociguat represents an effective long-term treatment option for PAH, either as a standalone therapy or in combination with endothelial receptor antagonists and prostanoids [16].

Racecadotril

Racecadotril is a neprilysin inhibitor (NEPI) that functions by blocking the action of neprilysin, a metalloproteinase enzyme responsible for deactivating various vasodilator peptides, including natriuretic peptides, bradykinin, substance P, adrenomedullin, and vasoactive intestinal peptides, as well as vasoconstrictor peptides like angiotensin 1 and 2 and endothelin-1 [40,41]. In preclinical models, a combination of a phosphodiesterase 5 inhibitor and a NEPI has been used to enhance natriuretic peptide bioactivity, amplify cGMP signaling, and ameliorate the structural and hemodynamic abnormalities specific to PAH [13]. Racecadotril is licensed for the treatment of secretory diarrhea and has previously been administered in trials with single doses ranging from 2 g up to three months for the management of acute diarrhea without causing significant side effects [42].

A randomized, double-blind, placebo-controlled proof-of-concept trial was designed to assess its safety and efficacy in patients with stable PAH who were already receiving PDE-5i therapy. In this trial, 21 patients were randomized into either the racecadotril group or the placebo group. The trial involved the evaluation of acute hemodynamic and biochemical effects after a single dose of racecadotril or a placebo, followed by a 14-day assessment of safety and effectiveness. The primary endpoint in both phases was the maximum change in circulating atrial natriuretic peptide (ANP) concentration, with secondary outcomes including measurements of pulmonary and systemic hemodynamics and mechanistic biomarkers [13]. The study concluded that the administration of racecadotril (100 mg) led to a 14% reduction in PVR, a 79% increase in plasma ANP concentration, and a 106% increase in plasma cGMP levels acutely. A 14-day treatment with racecadotril (100 mg three times a day) resulted in a 19% increase in plasma cGMP levels. Importantly, there were no significant side effects noted in either treatment arm. These results suggest that racecadotril may have therapeutic potential in the context of PAH; however, larger prospective clinical trials are needed to further evaluate its effectiveness [13].

## Conclusions

PAH is a severe, progressive, and life-limiting disease, despite the range of treatment options currently available. The FDA-approved therapies aim to restore the balance between vasodilators and vasoconstrictors, reduce vascular remodeling, and improve endothelial cell function. These treatments have led to increased survival rates, enhanced functionality, and better quality of life for patients. However, none of the current therapies offer a cure for PAH, and it remains a fatal condition. Therefore, there is an ongoing need for new and improved therapeutic options.
